# Supplementary material for: Predicting visual function by interpreting a neuronal wiring diagram
Source: Nature. 2024 Oct 2;634(8032):113–23. doi: 10.1038/s41586-024-07953-5 (PMC11446822; doi:10.1038/s41586-024-07953-5)
Supplement: Supplementary file 6 — CRF and ERF predictions for individual TmY4 and TmY9 cells. Analogous to Supplementary Data 3, but for TmY target types. Shown are the top four monosynaptic pathways, the strongest pathway passing through each of the top ten intermediary types (ranking from Extended Data Fig. 7), and the trisynaptic pathway Tm1–TmY–Dm3–TmY (see the section entitled Prediction of spatial normalization). [file 41586_2024_7953_MOESM6_ESM.zip › DataS4/TmY9qΓèÑ/720575940626973715.pdf]

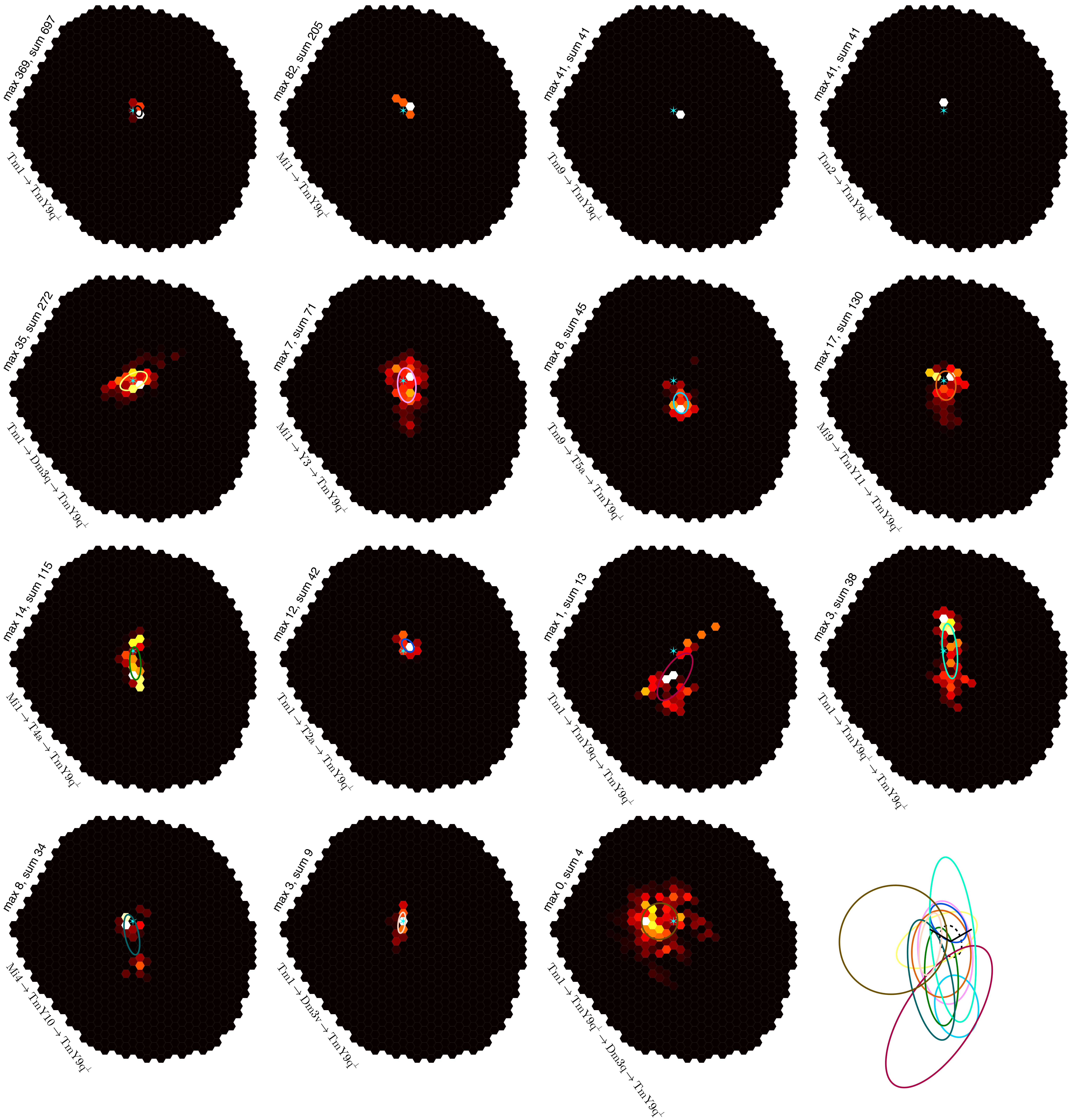

max 369, sum 697
Tm1 → TmY9q$^{\perp}$
max 82, sum 205
Mi1 → TmY9q$^{\perp}$
max 41, sum 41
Tm9 → TmY9q$^{\perp}$
max 41, sum 41
Tm2 → TmY9q$^{\perp}$
max 35, sum 272
Tm1 → Dm3q → TmY9q$^{\perp}$
max 7, sum 71
Mi1 → Y3 → TmY9q$^{\perp}$
max 8, sum 45
Tm9 → T5a → TmY9q$^{\perp}$
max 17, sum 130
Mi9 → TmY11 → TmY9q$^{\perp}$
max 14, sum 115
Mi1 → T4a → TmY9q$^{\perp}$
max 12, sum 42
Tm1 → T2a → TmY9q$^{\perp}$
max 1, sum 13
Tm1 → TmY9q → TmY9q$^{\perp}$
max 3, sum 38
Tm1 → TmY9q$^{\perp}$ → TmY9q$^{\perp}$
max 8, sum 34
Mi4 → TmY10 → TmY9q$^{\perp}$
max 3, sum 9
Tm1 → Dm3v → TmY9q$^{\perp}$
max 0, sum 4
Tm1 → TmY9q$^{\perp}$ → Dm3q → TmY9q$^{\perp}$
